# A case report of gadopentetate dimeglumine‐induced cardiac arrest: Resuscitation using extracorporeal membrane oxygenation

**DOI:** 10.1111/anec.13039

**Published:** 2023-02-03

**Authors:** Honglong Fang, Juan Chen, Jian Luo, Zhangping Li, Weiwen Zhang

**Affiliations:** ^1^ Department of Critical Care Medicine The Quzhou Affiliated Hospital of Wenzhou Medical University, Quzhou People’s Hospital Quzhou China; ^2^ Department of Laboratory Medicine The Quzhou Affiliated Hospital of Wenzhou Medical University, Quzhou People’s Hospital Quzhou China; ^3^ Department of Emergency Medicine The Quzhou Affiliated Hospital of Wenzhou Medical University, Quzhou People’s Hospital Quzhou China

**Keywords:** cardiac arrest, case report, extracorporeal membrane oxygenation, gadopentetate dimeglumine

## Abstract

Gadopentetate dimeglumine (Gd‐DTPA) is commonly used for enhancement in magnetic resonance imaging, but rarely causes serious adverse reactions. The patient presented in this report had a cardiac arrest and multiple organ dysfunction syndrome within a short time after administration of Gd‐DTPA. Immediately after receiving an intravenous injection of Gd‐DTPA, the patient felt nausea and chest tightness, and developed systemic erythema. He was successfully treated using veno‐arterial extracorporeal membrane oxygenation (ECMO) combined with continuous renal replacement therapy without any serious complications or neurological deficits. We report a patient who was successfully treated for Gd‐DTPA‐induced cardiac arrest with ECMO. Thus, ECMO may be an effective treatment for cardiac arrest secondary to anaphylaxis.

## Graphical abstract

We report a patient who was successfully treated for Gd‐DTPA‐induced cardiac arrest with veno‐arterial extracorporeal membrane oxygenation.

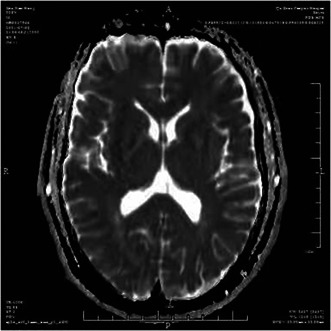

## 1 INTRODUCTION

A gadolinium‐based contrast agent (GBCA) is commonly used for enhancement in magnetic resonance imaging (MRI). The incidence of severe allergic reactions is <0.01% according to a literature review in 2021 (Bäuerle et al., 2021). One important risk factor for adverse GBCA reactions is a previous reaction to a contrast media (Ramalho & Ramalho, 2017). Carr concluded that severe allergic reactions to GBCA have an immunological etiology (Carr, 2016). It has also been reported younger patients are more prone to acute reactions (Okigawa et al., 2014).

The incidence of acute adverse reactions for gadopentetate dimeglumine (Gd‐DTPA), a commonly used GBCA, is approximately 0.14%, most of which are mild and only require clinical observation without specific treatment; cardiac arrests are infrequent (Abujudeh et al., 2010). Although extracorporeal membrane oxygenation (ECMO) has been widely used to treat in‐hospital cardiac arrest in patients undergoing non‐cardiac surgery, including cardiac arrest secondary to anaphylactic shock (Simons et al., 2015; Zhang et al., 2015), reports involving the treatment of Gd‐DTPA‐induced cardiac arrest are limited. Herein, we present a patient with Gd‐DTPA‐induced cardiac arrest who was effectively treated by emergency veno‐arterial ECMO.

## 2 CASE REPORT

A 58‐year‐old man weighing 70 kg was admitted to our hospital with penile cancer on April 27, 2021. Before undergoing a partial penectomy, an enhanced computed tomography (CT) with iohexol was obtained. A preoperative color Doppler ultrasound of the heart showed a small amount of mitral and tricuspid valve regurgitation. The medical history was benign and he was taking no medications. There was no history of drug allergies or adverse reactions to contrast‐enhanced CT. No medications were prescribed postoperatively at the time of hospital discharge on May 15, 2021.

A pelvic MRI was obtained on June 18, 2021 to evaluate inguinal and pelvic lymph node metastases after the penectomy. Immediately after receiving an intravenous injection of Gd‐DTPA at 15 ml/min, the patient was nauseous, had chest tightness, and developed systemic erythema. The imaging examination was promptly terminated and mechanical ventilation was initiated. According to the patient, this was the first exposure to Gd‐DTPA. Thus, the symptoms were considered to be an allergic reaction to Gd‐DTPA. The stat blood IgE level was 1215 (normal value <100). Three minute later, the patient was dyspneic and cyanotic, the heart rate was 55 bpm, and the blood pressure was 82/55 mmHg. Epinephrine (1:1000 [0.5 mg]) was injected intramuscularly, a bolus of lactated Ringer's solution was infused intravenously, and oxygen was administered via tracheal intubation. The patient developed ventricular fibrillation 8 min later. Bidirectional wave defibrillation (200 J) was delivered and cardiopulmonary resuscitation (CPR) commenced with chest compressions. Epinephrine (1 mg) was administered intravenously every 3 min. The cause of the in‐hospital cardiac arrest was severe anaphylactic shock. Spontaneous circulation was not restored after 40 min. There are numerous reports of patients with anaphylactic shock and cardiac arrest caused by other drugs who were rescued by ECMO, thus ECMO was initiated. The left femoral artery and venous ECMO was established within 13 min (21Fr access cannulation and 17Fr return). Heparin anticoagulation was not used due to a coagulopathy after prolonged CPR and severe gastrointestinal bleeding (stress ulcer confirmed by gastroscopy).

To obtain a mean arterial pressure ≥65 mmHg, the ECMO flow rate was initially set at 3.0 L/min; however, due pipe jitter the ECMO flow rate only reached 2.0 L/min, at which time the blood pressure was only 63/51 mmHg, so high doses (2.0 μg/kg/min) of norepinephrine and epinephrine were administered to maintain a mean arterial pressure of ≥65 mmHg. During this period, the patient had circulatory instability (left ventricular ejection fraction = 15%) with pulmonary edema and metabolic acidosis. The arterial blood gas results were as follows: pH, 7.086; bicarbonate ion (HCO_3_), 12.3 mmol/L; standard base excess (SBE), −17.1 mEq/L; and lactate, 19 mmol/L.

After 1 h on ECMO, ventricular fibrillation persisted despite two attempts at electrical defibrillation. Continuous renal replacement therapy (CRRT) combined with large amounts of plasma and albumin (i.e., heparin‐free anticoagulation strategy) were administered to stabilize the circulation, while reducing capillary exudation. Several hours later, the lactic acid and vasoactive drugs were progressively decreased. Electrical defibrillation was attempted again, which restored a spontaneous heart rhythm (128 beats/min) after 4 h of ECMO. The ECMO flow rate was set at 1.8 L/min. The doses of norepinephrine and epinephrine were 2.0 and 1.5 μg/kg/min, respectively. The blood pressure was 97/92 mmHg. The arterial blood gas results were as follows: pH, 7.43, HCO_3_, 19.4 mmol/L; SBE, −3.5 mEq/L; and lactate, 17 mmol/L. After 12 h on ECMO with a flow rate of 2.5 L/min, the norepinephrine and epinephrine doses were 0.5 and 0.5 μg/kg/min, respectively. The blood pressure was 146/105 mmHg. The arterial blood gas results were as follows: pH, 7.33; HCO_3_, 17.4 mmol/L; SBE, −7.2 mEq/L; and lactate, 15 mmol/L. After 24 h on ECMO with a flow rate of 2.0 L/min, the epinephrine dose was 0.5 μg/kg/min. The blood pressure was 118/63 mmHg. The arterial blood gas results were as follows: pH, 7.46; HCO_3_, 27.3 mmol/L; SBE, −3.7 mEq/L; and lactate, 2.9 mmol/L. Forty‐eight hours after ECMO, the patient had recovered completely without any serious complications or neurological deficits, and was discharged on hospital day 10. No further immunological testing was performed. There was no allergy‐related discomfort prior to hospital discharge and no discomfort at the subsequent outpatient follow‐up evaluation.

## 3 DISCUSSION

GBCA is considered to be very safe in clinical practice (Abujudeh et al., 2010; Zhang et al., 2015). Use keywords as below to search on PubMed, dated until June 28, 2022: “GBCA AND acute adverse reaction,” “gadopentetate dimeglumine AND acute adverse reaction,” “gadobutrol AND acute adverse reaction,” “gadoterate meglumine AND acute adverse reaction,” “gadopentetate dimeglumine AND cardiac arrest.” After reading the titles and abstracts, the reports about severe or serious acute reactions were selected and summarized in Table 1. As shown in Table 1, the incidence of GBCA (including Gd‐DTPA)‐associated acute severe reactions is 0.003%–0.04%. Only five patients with cardiac arrest due to the use of GBCA have been reported as of June 28, 2022, and epinephrine and CPR are common treatment strategies for such cases (highlighted in yellow, Table 1); ECMO was not discussed as a rescue strategy. Among the five patients with cardiac arrest, four were revived and one died; however, the rescue details were not disclosed (highlighted in yellow, Table 1). Our patient had a dramatic I‐type allergic reaction (IgE‐mediated) to Gd‐DTPA, which is similar to the report by Schiavino et al. (Schiavino et al., 2003). Our patient rapidly progressed to ventricular fibrillation 8 min after Gd‐DTPA use. A spontaneous circulation was not restored, despite external chest compressions, epinephrine injections, and defibrillation. Without ECMO support, the outcome would most likely have been death.

**TABLE 1:** Literature review of GBCA‐associated acute severe reactions

Author	Rate of acute severe reactions	Acute severe reactions	Immediate treatment	Recommended
Hojreh et al., 2020	2/8156; 0.02%	Tonic–clonic convulsion, intermittent respiratory complaints	Only non‐contrast	a fast‐track allergy work‐up
Tanaka et al., 2019	Case report	Acute coronary syndrome (Kounis syndrome)	Administration of nitroglycerine, atropine sulfate, and hydrocortisone	Be vigilant about cardiovascular patients
McDonald et al., 2019	3/94109; 0.003%	Allergic‐like severe reactions	Hospitalization; two patients required subsequent extracorporeal membrane oxygenation support	n/a
McDonald et al., 2019	3/39138; 0.008%	Allergic‐like severe reactions	Hospitalization	n/a
Uhlig et al., 2019	24/72839; 0.033%	Arrhythmias, renal failure, resuscitation, severe allergic reaction	n/a	n/a
Kato et al., 2019	Case report	Acute respiratory distress syndrome	Admitted to the intensive care unit, artificial respiration management and adrenaline and steroid administration	n/a
Granata et al., 2016	4/10608; 0.04%	One severe respiratory distress, an episode of progressive angioedema, and one of arrhythmia, consisting in supraventricular tachycardia; one severe respiratory distress	Transferred to the intensive care unit and discharged after 24 h of observation.	Evaluate the data in relation to the use of accurate premedication
Bruder et al., 2015	2/37788; 0.005%	Suffered anaphylactic reactions that were graded as severe events due to the combination of bronchospasm and profound hypotension	Treated with adrenaline, steroids and antihistamines.	n/a
Lee et al., 2019	Case report	Acute respiratory distress syndrome	Methylprednisolone and bilevel positive airway pressure ventilation, discharged in stable condition on day 6.	Document severe allergic reaction or idiosyncratic reaction
Abujudeh et al., 2010	2/32659; 0.006%	One case (male) had a seizure, and became unconscious; Another (female) had acute respiratory distress, followed by cardiopulmonary arrest with pulseless arrhythmias	Male case: transferred to the emergency department and discharged; Female case: cardiopulmonary resuscitation, transferred to the emergency department and discharged;	n/a
Turk et al., 2018	Case report	Tonic–clonic generalized seizure	Acute management with antiepileptics, cerebrospinal fluid irrigation by external ventricular drainage helped the patient's improvement.	n/a
Kalogeromitros et al., 2007	Case report	Bronchospasm and acute urticaria with diffuse giant pruritic plaques	Acute treatment	skin testing seems to be a precious diagnostic tool
Guru et al., 2016	Case report	Severe pulmonary edema	Emergency extracorporeal membrane oxygenation resuscitation	n/a
Zlojtro et al., 2013	Case report	Kounis syndrome	Epinephrine (1 mg) intramuscular, chloropyramine chloride (10 mg) IV, methylprednisolone (125 mg) IV and 0.9% NaCl (1000 ml)	Contrast agents may cause Kounis syndrome
Virtos et al., 2015	Case report	Anaphylactic reaction and cardiac arrest, with multiple complications, in particular a critical illness polyneuropathy and a severe ischemic colitis	Recovered after 16 min of cardiopulmonary resuscitation, 12 mg of intravenous titrated epinephrine and alkalinization. A few minutes after resuscitation, the patient suffered a second cardiac arrest and recovered in 4 min. After this second episode, she was transferred to the intensive care unit.	Some people may have severe Anaphylactic reaction to GBCA
Unal & Arslan, 1999	Case report	Cardiac arrest	n/a	n/a
Prince et al., 2011	4/158796, 0.003%	The first case was a case of cardiac arrest and death	The human who died was with a history of gastrointestinal bleeding; underwent liver MRI; he was noted to be red, dyspneic, and unable to cooperate with breath‐holding; he then became unresponsive and could not be revived	This study raises the possibility that non‐ionic linear gadolinium‐based contrast agents and gadopentetate dimeglumine may have fewer severe immediate adverse events compared with gadobenate dimeglumine
Prince et al., 2011	4/158796, 0.003%	The second case: a 65‐year‐old male outpatient with meningioma was initially itchy and shaking, then became unresponsive. Wide complex tachycardia (120 beats per minute), hypotension (60/30 mmHg), and dyspnea (30 breaths per minute) was noted	The patient was intubated and responded to intravenous dopamine, normal saline, D50, calcium chloride, and lidocaine. After transferring to the emergency department and later to the coronary care unit, the patient stabilized, was extubated, and was discharged home 2 days later.	This study raises the possibility that non‐ionic linear gadolinium‐based contrast agents and gadopentetate dimeglumine may have fewer severe immediate adverse events compared with gadobenate dimeglumine
Prince et al., 2011	4/158796, 0.003%	The third case was a 60‐year‐old male inpatient who complained of a metallic taste, then became unresponsive with tachycardia (150 beats per minute), hypotension (70/40 mm Hg), and cyanosis (O_2_ saturation = 70%).	Successfully revived the patient with epinephrine, IV fluids, diphenhydramine, solumedrol, and oxygen.	This study raises the possibility that non‐ionic linear gadolinium‐based contrast agents and gadopentetate dimeglumine may have fewer severe immediate adverse events compared with gadobenate dimeglumine
Prince et al., 2011	4/158796, 0.003%	The fourth case was a 9‐year‐old boy who became combative during an MRI with urticaria, wheezing, and desaturation (oxygen saturation in the 80s) occurring after injecting 10 ml gadoteridol	He responded to epinephrine, albuterol, diphenhydramine, and decadron; after stabilizing and recovering from anesthesia, the patient was discharged home	This study raises the possibility that non‐ionic linear gadolinium‐based contrast agents and gadopentetate dimeglumine may have fewer severe immediate adverse events compared with gadobenate dimeglumine
Jordan & Mintz, 1995	Case report	She became very short of breath after injection; pulse rate decreased and blood pressure increased. Full cardiopulmonary arrest ensued; finally, she was died	Epinephrine (0.3 mg, 1 g/1000 ml) was administered subcutaneously, as no IV site was immediately available; atropine (1 mg) IV was also given; the patient did not respond to these measures, became progressively diaphoretic and cyanotic, and was soon in full respiratory arrest; full cardiopulmonary arrest ensued, and cardiopulmonary resuscitation was initiated; resuscitative efforts were continued in the emergency department but were unsuccessful; autopsy showed severe bullous emphysema; focal, moderately severe (60%) atherosclerosis of the left coronary artery was present	The presumed cause of death is an anaphylactic reaction with associated bronchospasm; radiologists and MR imaging personnel should therefore remain alert to the possibility of severe, rarely fatal, reactions to gadolinium‐based contrast agents, especially in patients with a history of asthma or other chronic respiratory disease.

ECMO has been shown to be effective in providing circulatory support to patients in cardiogenic shock or cardiac arrest by providing respiratory support alone, respiratory and right ventricular support, or full cardiopulmonary support (Ratnani et al., 2018; Simons et al., 2015). Early ECMO initiation provides benefits, such as delivering appropriate oxygenation and/or tissue perfusion, and providing lung and heart rest. The use of ECMO in the early stages of trauma may increase the risk of bleeding due to systemic heparin anticoagulation, especially in patients with intracranial or active systemic bleeding (Wang et al., 2020). Therapeutic heparin may increase the risk of bleeding. Patients can be successfully managed on heparin‐free ECMO without worsening the degree of bleeding (Amos et al., 2019). Abujudeh et al. used ECMO to assist a patient with cardiopulmonary arrest due to GBCA use and the patient recovered (Abujudeh et al., 2010). ECMO was used in our patient immediately when the spontaneous cardiac rhythm could not be restored after routine, high‐quality CPR.

CRRT is indicated for patients with acute kidney injury who are hemodynamically unstable, thus allowing precise volume control, correction of metabolic disturbances, and stable acid–base and electrolyte correction (Karkar & Ronco, 2020). In addition, CRRT was implemented in a patient who was diagnosed with an acute kidney injury (AKI) to correct metabolic disturbances and control volume (Karkar & Ronco, 2020). In addition, CRRT may decrease internal contrast media levels, thereby alleviating allergic reactions (Song et al., 2021).

An expedited allergy work‐up with skin testing and assessment of premedication data may help prevent an allergic reaction to GBCA, as shown in Table 1. The reported patients with cardiac arrest due to GBCA are limited, thus the underlying mechanism and effective prevention strategies have not been established. Patients with atherosclerosis, cardiovascular disorders, or chronic respiratory diseases warrant a thorough evaluation (Table 1).

## 4 CONCLUSION

In conclusion, we report the first case of successful treatment of Gd‐DTPA‐induced cardiac arrest using ECMO. Our results suggest that ECMO may be an effective treatment for cardiac arrest secondary to anaphylaxis. Moreover, an expedited allergy work‐up with assessment of premedication data and skin testing may be helpful to screen patients who are allergic to GBCA. Patients with atherosclerosis, cardiovascular disorders, or chronic respiratory diseases warrant special attention.

## AUTHOR CONTRIBUTIONS

Conception and design of the research: Fang HL, Chen J, Li ZP. Acquisition of data: Fang HL,Chen J, Zhang WW. Analysis and interpretation of the data: Luo J, Zhang WW, Fang HL. Statistical analysis: Luo J, Zhang WW. Writing of the manuscript: Fang HL, Chen J, Li ZP. Critical revision of the manuscript for intellectual content: Li ZP. All the authors have approved the final draft for submission.

## FUNDING INFORMATION

This case report was supported by Project of Zhejiang Provincial Department of Health (2021KY1192) and Quzhou Bureau of Science and Technology (2022K71).

## CONFLICT OF INTEREST

The authors declare no conflicts of interest, financial, or otherwise.

## CONSENT

The patient has consented to submission of the case report to the journal.

## ETHICS STATEMENT

This study was approved by the Institutional Ethics Review Board (No. Ethics‐Y‐2020‐05‐06). Informed written consent was obtained from the patient.

## Data Availability

The data that support the findings of this study are available on request from the corresponding author.
